# Social Responsibility: A New Paradigm of Hospital Governance?

**DOI:** 10.1007/s10728-012-0206-3

**Published:** 2012-04-06

**Authors:** Cristina Brandão, Guilhermina Rego, Ivone Duarte, Rui Nunes

**Affiliations:** Department of Bioethics, Faculty of Medicine, University of Porto, Estrada da Circunvalação 9925, 4250-150 Porto, Portugal

**Keywords:** Corporate governance, Health care, Hospital, Social responsibility

## Abstract

Changes in modern societies originate the perception that ethical behaviour is essential in organization’s practices especially in the way they deal with aspects such as human rights. These issues are usually under the umbrella of the concept of social responsibility. Recently the Report of the International Bioethics Committee of UNESCO on Social Responsibility and Health has addressed this concept of social responsibility in the context of health care delivery suggesting a new paradigm in hospital governance. The objective of this paper is to address the issue of corporate social responsibility in health care, namely in the hospital setting, emphasising the special governance arrangements of such complex organisations and to evaluate if new models of hospital management (entrepreneurism) will need robust mechanisms of corporate governance to fulfil its social responsiveness. The scope of this responsible behaviour requires hospitals to fulfil its social and market objectives, in accordance to the law and general ethical standards. Social responsibility includes aspects like abstention of harm to the environment or the protection of the interests of all the stakeholders enrolled in the deliverance of health care. In conclusion, adequate corporate governance and corporate strategy are the gold standard of social responsibility. In a competitive market hospital governance will be optimised if the organization culture is reframed to meet stakeholders’ demands for unequivocal assurances on ethical behaviour. Health care organizations should abide to this new governance approach that is to create organisation value through performance, conformance and responsibility.

## Introduction

Changes in modern societies were driven by several factors, namely economic and cultural globalization, scientific and technological progress, increased access to information, or the acknowledgement of consumers’ rights. All these changes originate the perception that ethical behaviour is essential in organization’s practices especially in the way they deal with aspects such as human rights. These issues are usually under the umbrella of the concept of “social responsibility”. By social responsibility it is meant that organizations can meet its fundamental goals of accomplishing a particular public endeavour or of increasing shareholders’ profits, but at the same time fulfilling other important objectives namely with regards the satisfaction of stakeholders’ interests. Sometimes “social responsibility” is also associated with environmental concerns and the protection of the commonwealth of life [4].

Recently this concept of social responsibility has been addressed in the context of health care delivery suggesting a new paradigm in hospital governance. Namely the Report of the International Bioethics Committee of UNESCO on Social Responsibility and Health, commenting on the right to health care access, states that “What is at stake is a fundamental right, together with the awareness of a limit of attainability. The novelty and the importance of this article is that it explicitly widens the concept of social responsibility, applying it not only to the private sector but also to the public domain. That’s why the task of social responsibility is to be shared by the private sector and States and governments, which are called to meet specific obligations to the maximum of the available resources in order to implement and progressively achieve the full realization of this right.” [27]. Indeed, there is a growing conviction that the deliverance of health care, just like other aspects of social life, should be driven in accordance with universal ethical principles, respecting the human being and its fundamental rights [19]. The concept of social responsibility implies that a shared vision of the common good is universally accepted among health care professionals, other stakeholders and the overall social matrix.

The objective of this paper is to address the issue of corporate social responsibility in health care, namely in the hospital setting, emphasising the special governance arrangements of such complex organisations and to evaluate if new models of hospital management (entrepreneurism) will need robust mechanisms of corporate governance to fulfil its social responsiveness.

## Social Responsibility and Social Responsiveness

The number of organisations that embrace a socially responsible conduct is becoming increasingly larger, meaning that citizens, and investors, are deeply aware that profit and ethical values (namely with regards protection of human rights) are not incompatible [2]. Embracing a socially responsible conduct can be seen as strategic in a global market, contributing to the competitiveness of a company and protecting its external image. It implies an adaptive effort of many corporations due to market rules and public expectations. Involving the public in such decisions will lead to the promotion of universally shared values namely with regards the intervention of large companies and banks in nations were law is difficult to apply. However, social responsibility should be implemented voluntarily out of beneficence values and not only, as suggests Amir Barnea, for the private benefit of the managers to improve their own reputation [1].

The reconciliation between profit and social responsibility of corporations leads us to a new market that accounts for more than 10 % off all the investments at the New York Stock Exchange. At the beginning of 2010, professionally managed assets following Social Responsible Investments strategies stood at $3.07 trillion, a rise of more than 380 % from $639 billion in 1995, the year of the Social Investment Forum Foundation’s first Trends Report [25]. Indeed, the empowerment of responsible citizens is nuclear and fundamental. For instance, public and democratic accountability would be a determinant in the bioevaluation of new technologies like genetic engineering in animals and food (transgenic).

There is a latent tension between social responsibility and profit making because traditional business ethics determines quite clearly that the business of business is business, which implies that the main goal of private corporations is to increase the profits of its shareholders. In this perspective any use of corporations’ resources to other goals than profit making would be unethical because that use is not legitimated by shareholders. However, in the last decade there is a growing social awareness that profit is a necessary condition but not a sufficient one. There are other laudable goals that major corporations should pursue besides profit. Moreover, shareholders consent could be presumed if those goals are clearly stated in the corporation mission. It follows that the concept and practice of social responsibility could be easily commended to a profit making company because it would not only increase its external image but, more importantly, shareholders in a modern society would know that a specific corporation manages its internal and external operations with other goals beyond profitability.

Also, in developed societies, integration of social responsibility into business practice is an expected policy and there are many good examples of this change. Both in local communities as well as at a global level social responsibility is a practice expected by many shareholders and by most stakeholders. There are plenty of good examples of social responsibility projects in local communities, such as in education, in social inclusion or even in cultural areas.

In health care, corporate social responsibility means that there is an ethical obligation that requires hospitals and other organizations to do something beneficial in issues such as delivering quality health care to everyone who is entitled to it. It is not easy to practice social responsibility because the satisfaction of some stakeholders’ interests may be opposed to the fundamental goal of most health care systems. In spite of this paradox, that can originate some difficulty in the management of health care organizations, many hospitals (for profit and not for profit) have applied the concept of social responsibility through explicit interventions in management decisions. To accomplish this ideal, companies should define objectives (the mission) and social programmes that integrate ethical principles not only in strategic planning but also in its daily activity.

Therefore, social responsibility is concerned with the way a particular organisation manages its internal operations, as well as the impact of its activities in the social environment. From this perspective a distinction can be drawn between passive and active social responsibility (Table 1).

**Table 1:** Types of hospital social responsibility

*Passive social responsibility*
1. Creating wealth and promoting employment
2. Protecting the investment of all shareholders (namely the government in public hospitals)
3. Protecting the interests of all stakeholders
4. Respecting human rights
5. Abstention of environmental damage (namely in dealing with toxic waste)
6. Abiding to the law
*Active social responsibility*
1. Implementing ethical codes of conduct
2. Promoting reverse discrimination policies (affirmative action)
3. Public accountability of management decisions and performance indicators
4. Protecting animal interests (namely in research)
5. Contributing actively for environmental protection
6. Engaging in national or international solidarity programs

What is, then, the scope of this responsible behaviour? Put quite simply, passive social responsibility only requires hospitals to fulfil its social and market objectives, in accordance to the law (national and international) and general ethical standards, even if they could do more good or improve society by other set of goals. Respecting human rights is the paradigm of social responsibility and should be a goal of any health care organization. Non-discriminatory policies at work or protecting privacy rights are examples of such a responsible behaviour. Policies that protect society against toxic waste or prevention of animal damage in research are also within the scope of this concept. This type of social responsibility also includes aspects like lawful behaviour, abstention of harm to the environment or the protection of the interests of all the stakeholders enrolled in the deliverance of health care.

Active social responsibility requires organizations—namely hospitals—to do something beneficial (out of beneficence duties) and not only abiding to the law or to general ethical principles. It follows that interest and values of all stakeholders are taken into consideration [12]. Again the Report of the International Bioethics Committee of UNESCO on Social Responsibility and Health suggests that “Social responsibility should be understood as being part of what has traditionally been called moral obligations. These obligations cannot be imposed by others or by the State. The difference between legal and moral obligations does not imply that the latter are less important. It simply implies that there is no legal coercion to fulfill them. Nonetheless, the more significant the consequences for failure to conform to these norms, the greater is the moral obligation to do so. This is especially so when we consider the duties deriving from a fundamental right.”

The existence of a right to health care, as a positive social right, emphasises this perspective and the need for hospital active social responsibility. It implies that organizations contribute with its resources or skills to the common good. For instance, the implementation of ethical conduct codes, affirmative action policies at the workplace (reverse discrimination of minority groups) or the active contribution in promoting the environment, are good examples of this kind of social responsibility. Re-interpreting the concept of social responsibility means that an organization should not only fulfil its economic and legal obligations but also actively contribute to the social good. A good example of an ethical behaviour insofar as the common good is at stake is the fact that many teaching hospitals have developed guidelines for animal welfare in research and have even created specific ethics committees for dealing with these issues.

In short, hospitals and other health care organisations should abide to common ethical principles that include aspects like:

1. Avoiding precarious labour and supporting flexible labour policies, namely with regards women during the breast-feeding period;
2. Creating assistance services for the handicapped;
3. Creating assistance services to satisfy spiritual or religious needs;
4. Supporting programs of social well-being and solidarity;
5. Implementing marketing strategies that abide to ethical standards;
6. Treating with caution toxic, poisonous residues, potentially dangerous to humans and animals;
7. Protecting animal rights;
8. Preserving the environment;
9. Promoting policies that enrol in the decision-making process specific groups of patients and Non-Governmental Organisations (NGOs).

It is disputable however the nature of social responsibility in the sense of who are relevant stakeholders and what their interests are. Also it can be questioned what is the role of investors, employees, customers, tax payers, as well as more impersonal bodies such as the environment and how their competing interests are fairly balanced. Corporate social responsibility is accomplished only if a new model of corporate governance is implemented. New governance arrangements mean that there are internal mechanisms of control that take into consideration all stakeholders interests and valid claims. Public and stakeholders’ accountability is also an imperative of good corporate governance so that any decision that goes beyond the managers/shareholders contract is not an arbitrary one but is taken in accordance with societal expectations. A dual board with a formal overseeing authority of the executive board is also a fundamental step towards good social performance.

National and international law related to social responsibility is already in practice, but law by itself is insufficient to promote such an ethical behaviour. A different approach is needed because ethical rules do not exist in many instances. Legislation is just a minimum that guides organizations conduct. But, many providers are willing to do more than this minimum demanded by law, requesting their certification of social responsibility under international norms—Social Accountability SA 8000 and ISO 26000. These are international norms that intend to implement better work conditions based in the principles of the International Labour Organisation, the United Nations Convention on Children’s Rights and the Universal Declaration of Human Rights.

**Fig. 1:**
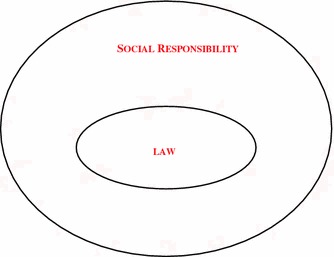
Interface between law and social responsibility

Legal regulation is a necessary condition for good corporate social performance but an insufficient one. In modern societies legislation and its application ensure the legality of management decisions. But there are many aspects of corporate performance that are beyond strict legality. For instance apparel industry is not legally required to engage in national or international solidarity programs for the elderly or migrant communities. But many do so. Law only serves to ensure that a corporation acts responsibly in the passive sense previously described but not in a proactive manner to do more than it is usually expected (Fig. 1).

There is a vast array of social goods towards which social responsibility strives. The values can be considered as universally shared: promoting freedom and equal opportunities or protecting the environment and the commonwealth of life are values that any corporation should pursue, although public discussion about specific programs should be promoted by any socially responsible organisation. In the health care setting there is no doubt whatsoever about the set of values that should be pursued. Equity in access, universal coverage, and efficient resource allocation is the ethical platform of modern health care systems. Again, public accountability of any management decision would legitimise it and would also promote public awareness in a more enlarged societal debate.

The concept of social responsibility means that it is not enough for a health care organisation to bet in the quality of its services, in competitive prices or in advanced technology, but it is also important to be respected by its customers, professionals and society at large [3]. Therefore, the adoption of a socially responsible conduct could be an important step for a hospital to increase its competitiveness and to protect its external image. For example, a social responsible hospital should analyse and find which way to treat or to discard safely a waste product that can contaminate the environment. It should be remembered that developed countries are responsible for 75 % of the world energy production and resource consumption and create a large proportion of toxic waste. But their level of pollution is relatively low due to severe laws, industrial concern about ecology and the relocation of polluting activities into emerging markets.

It is usually accepted that health care organisations that are socially responsible are more prone to be in favour of supporting local communities [30]. Also it is more likely that solidarity programs are put forward, because many hospitals are engaged in the organisation of social projects for the least advantaged, in the promotion of employment and in the implementation of reverse discrimination policies to protect cultural minorities. Lastly, self-regulation is promoted due to social pressure and financial investment.

The more an organisation has closer community links the more it will be possible to engage in social responsibility. Community linked management models adapt best to these practices because, in spite of its public nature, they appear to be more and more competitive and, therefore, will tend to focus on achieving added value, concentrate on the different stakeholders, emphasize result optimization and performance evaluation, as well as value innovation and entrepreneurism. Furthermore, attributes such as greater autonomy, a participative structure, a high degree of flexibility and openness, national and global coverage integrated in an interdependent network strengthens this governance model.

Corporate Social Responsibility in health care includes all involved agents namely pharmaceutical Industry [15]. However, due to the specificities of these corporations and notwithstanding the fact that in some areas (like orphan drugs) their social responsiveness should be optimized, this article will emphasize especially hospital social responsibility [13]. It seems clear that organisations, namely hospitals, need to balance conformance with performance and corporate responsibility. It is instrumental to meet stakeholders’ demands for unequivocal assurances on efficiency, ethical behaviour and value. Adequate corporate governance and corporate strategy are therefore the gold standard of social responsibility.

## Hospital Corporate Governance

**Fig. 2:**
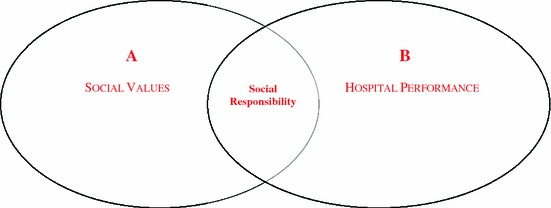
Drivers of social responsibility

Pressure from globalisation is leading to a redefinition of the social function of many health care organisations. Indeed, some of them have as a social goal delivering quality health care and as an economical goal to increase wealth and employment in a particular community and also to contribute to the development of new technologies. However, what is expected today from a hospital is also its contribution to a fairer society and a safer environment. Health care organisations trying to act in a socially responsible way should do what is right, minimizing the potential damage to the stakeholders (Fig. 2). Corporate governance is the operational paradigm of this dimension of social responsibility [10].

Indeed, health care organizations face new challenges in modern societies. The lack of economical sustainability of most health care systems led to the introduction of the new public management to guarantee the survival of the welfare state. The corporatisation of public hospitals was accomplished through many organizational changes, namely creating a split between purchasing and provision using output-based contracts for which private and public firms compete, giving managers’ greater autonomy and experimenting with incentive payments and other ways of improving worker productivity [14]. The introduction of the private finance initiative (PFI) perspective—in which a private group delivers health services (including sometimes clinical services) on a contractual basis—is sometimes thought to be another way to increase the value for money [26]. This transformation of many health care systems in Europe as elsewhere led to creation of robust mechanisms of regulation, to guarantee the pursuit of the public good [28]. It follows that a new organizational culture is deemed necessary to overcome the market failures that can arise with the entrepreneurialism in health care [18].

The concept of social responsibility emerges with reference to privately owned, profit making organisations, so it might be assumed that social responsibility is already incorporated into the governance of public health care provision. However, there is scope for some social intervention beyond strict statutory provision. Non-profit, publically owned corporations that are managed as private corporations (such as private finance initiative or corporate public hospitals) should also promote policies of social responsibility. Not only to avoid unethical practices such as cream skimming and induced demand (passive responsibility) but also to proactively promote activities such as implementing ethical codes of conduct for managers or engaging in solidarity or cultural programs. Also, public accountability of management decisions and performance indicators is an ethical and social imperative that usually is not obliged by law. This is another example of active social responsibility that should be implemented whatever the institutional nature of the health care organization.

The increase in corporate benchmarking indices and the growth of stakeholder activism in most economical activities makes it an imperative to increase its social responsiveness and health care organisations, such as hospitals, must also address the issues of balancing corporate governance with corporate strategy. This social responsiveness implies organizational changes in mechanisms, procedures, arrangements and behavioural patterns that collectively make health care organizations capable to respond to social pressures [5].

But it should remain clear that new governance strategies are in accordance with a framework of quality assurance based on the evaluation of structure, process and outcome and should try to reinterpret this perspective in light of the social responsibility of health care organizations. As stated by Avedis Donabedian [8] “Traditionally, in health care, quality assurance has been meant to apply predominantly, or even exclusively, to health care itself as provided directly to patients by legitimate health care practitioners. Removed one level, we include other services that directly affect the ability of practitioners to perform well, meaning such things as radiological, pharmaceutical, and laboratory services. In recent years, under the rubric of “total quality management”, it has become popular to include in the idea of quality assurance almost every function or activity of a health care organization or system, including such things as the performance of housekeeping crews, secretarial and clerical services, the billing and fee-collecting office, and so on. It is reasonable to say that the quality of the immediate environment of health care, including the managerial activities in it, have an influence of the quality of care—either directly, by influencing the performance of practitioners or indirectly, by influencing the convenience, comfort, or safety of patients.” It follows that in any health care organization quality assurance is a presupposition for adequate social responsibility.

In accordance with McSherry and Pearce [17] health care governance should unite three different elements of health service governance: corporate governance (management), clinical governance (clinical practice) and non-clinical supporting services (controls assurance). We will focus in corporate governance in health care organizations as we feel that good corporate governance is instrumental for clinical governance and controls assurance to succeed. Corporate governance is an evolving area and its development has been driven by the need to restore confidence in management decisions in liberal markets (Table 2).

**Table 2:** Principles of hospital corporate governance

1. Goals of hospital corporate governance: to increase its performance, to assure its social responsibility namely concerning the search for the common good, and to promote conformance with regards accountability arrangements in a fair and transparent way [9]
2. External controls
(a) *Public accountability*: Explicit, public detailed procedures for evaluating hospital performance with full public report (use reports, performance reports, compliance reports, consultants), global budgeting, fair grievance procedures (legal, non-legal), and adequate privacy protection (adapted from [7])
(b) *Stakeholders accountability*: External mechanisms of reporting, public disclosure of the processes and rationale adopted in management, external audit, financial account and annual report (published at internet)
3. Internal controls
(a) *Self-regulation*: Internal audits, ethical codes and disclosure of directors’ performance and remuneration
(b) *Board*: Unitary versus dual board, mechanisms of appointment to the board, performance evaluation (adapted from [16])

Indeed, health care has traditionally been delivered in many countries through public organisations and the search for equity in access and distribution has been the main goal of public providers of most public systems. In the last decades the costs of health care, including essential public health functions, have increased steadily due to biomedical research (e.g., genetics), emerging diseases (e.g., AIDS), medical malpractice and increased life expectancy. Increasing efficiency became then another driver of most health care systems. The growing dilemma of most developed countries is how to assure that equity, efficiency and quality are simultaneously delivered to the population. The balance between those values is frequently difficult to accomplish but, nevertheless, public providers try to be responsive to public expectations.

Efficiency is frequently addressed by the introduction of entrepreneurialism in health care but this evolution can affect equity as well as quality standards (quality shading). Therefore there has been a growing concern over aspects of quality assurance of health care organizations. The delivery of care is progressively fuelled by concepts such as evidenced based medicine, controls assurance, clinical governance all trying to achieve a continuous improvement in health care and a satisfying risk management control. New governance arrangements of health care originations, such as central hospitals, are therefore necessary to reinterpret the meaning of quality care beyond classical definitions centred only in the system (structure), the organised activities (process), and the final outcome. The modern hospital should measure the outcome also by the impact of the hospital as a complex organization in the environment, particularly in the surrounding society because, as claimed by Cecile Renouard, an anthropological perspective of social responsibility is more prone to promote sustainable human development than a strict utilitarian one [22].

The main drivers of corporate governance are to increase the performance of the organisation and assuring its social responsibility and responsiveness namely concerning the search for the common good, and to promote conformance with regards accountability arrangements in a fair and transparent way. To accomplish these purposes, a complex system of external and internal controls is usually developed. Public accountability is the genetic print of good corporate governance through the existence of public detailed procedures for evaluating the health care organization activity, with full public report, global budgeting, fair grievance procedures, adequate privacy protection, external audit, financial account and annual report [7], as well as internal audits, ethical codes, disclosure of directors’ performance and remuneration and performance evaluation. For instance, codes of ethics are an integrant part of the ethics program of most modern hospitals. This instrument is usually supplemented by other measures such as the existence of an ethics committee and ethical training [24]. However, a hospital code of ethics should promote the empowerment of all health care professionals in order to develop even further professional ethical norms and obligations [31].

In public health care systems—both Beveridge and Bismarck systems alike—the entrepreneurialism needs robust governance arrangements because corporatisation aims to increase efficiency and the value for money [21]. As stated by Preker and Harding “with increasing frequency, autonomization and corporatization are being considered and applied to improve performance of publicly run health services, similar to recent innovations in organizational reform elsewhere in the public sector” [20]. Entrepreneurism led to many different models and it is expected that all of them fulfil the values embraced by modern democracies but a participative hospital management model makes the process of accountability even more visible. Indeed, trust is a fundamental aspect of the moral treatment of stakeholders within an organization-stakeholder relationship [11].

In a competitive market, good hospital governance will prevent unethical practices such as cream-skimming and induced procurement of care practices strongly regulated by independent regulatory agencies [29]. Other goals could also be optimised if the organization culture is reframed to meet stakeholders’ demands for unequivocal assurances on ethical behaviour. Health care organizations should abide to this new governance approach that is to create organisation value through performance, conformance and responsibility.

## Conclusion

The expansion of the concept of social responsibility from the private sector to public organizations is a challenge and an opportunity that should be clearly embraced [23]. Social responsibility and social responsiveness in health care imply both a new social dimension of care as well as new organizational patterns of hospitals and other health care organizations. Indeed, bad governance can destroy an organisation, in particular a hospital or other health care organization. It is also true that good corporate governance should be balanced with effective strategic management if sustainability is to be achieved.

Corporate social responsibility in health care does not apply only to hospitals and other health care organizations, but also to multi-national companies namely pharmaceutical companies. In this context social responsibility has a wider field of intervention because issues such as human rights, gender equality, child labour, and environment protection have different meanings in different cultures. Nevertheless in a globalised culture and economy, social responsibility is a concept that transcends local moralities and should promote transnationally values that are in accordance with international conventions of human rights and environmental protection. Namely the pharmaceutical industry could reinterpret the concept of social responsibility by allowing the access of particularly vulnerable populations of the underdeveloped world to medicines that are lifesaving to many people but that particular circumstances make it very difficult to provide in a fair and systematic way.

If economic freedom determines and is determined by the rules of the competitive market, there is no doubt that in modern societies—governed by a sense of citizenship and civic consciousness—there is a search for social responsible organizations [6]. Hospitals can and should be the first ones to follow this path, helping to shape public policies and consumer behaviour, because incorporating ethical concerns presupposes the assumption that organisations need to balance conformance with performance and corporate responsibility. Health care organisations should try to understand their mission in a global society, promoting shared values and common ethical principles in new patterns of hospital governance.
